# Botanical origin and characterization of monofloral honeys in Southwestern forest of Ethiopia

**DOI:** 10.1002/fsn3.2453

**Published:** 2021-07-14

**Authors:** Admassu Addi, Tura Bareke

**Affiliations:** ^1^ Holeta Bee Research Center Oromia Agricultural Research Institute Holeta Ethiopia

**Keywords:** honey, honey quality, melissopalynology, monofloral honey

## Abstract

The study was conducted to identify and characterize the monofloral honey types found in the Gesha‐Sayilem forest. A total of 15 honey samples were collected during the honey flow seasons. For honey pollen analysis, the method recommended by the International Commission for Bee Botany and harmonized methods of the International Honey commission were used. Data were analyzed using one‐way ANOVA, PCA, and Pearson correlation coefficients. Three monofloral honey types were identified, such as *Schefflera abyssinica* honey, *Croton macrostachyus* honey, and *Vernonia amygdalina* honey types. The mean moisture content of the honey samples of *Vernonia amygdalina* honey was 18.3 ± 1.02%, that for *Schefflera abyssinica* honey 18.1 ± 1%, and 21.2 ± 1.05% for *Croton macrostachyus* honey. The HMF value of the *Vernonia* honey ranged from 1.1 to 1.3 mg/kg, with a mean value of 1.2 ± 0.1 mg/kg; that of *Schefflera abyssinica* honey ranged from 2.2 to 2.5, with a mean value of HMF 2.3 ± 0.15; and that of *Croton* honey ranged from 2.4 to 2.6 mg/kg, mean value of 2.56 ± 0.15 mg/kg. There was a significant difference in the free acid content of honey samples due to the botanical origin of honey and sampling locations (*p* < .05). The electrical conductivity of honey samples in the Gesha‐Sayilem forest was found within an international range, with a maximum limit of 0.8 mS/cm for most nectar honey. There was a significant strong correlation between proline, free acid, and sucrose. Moisture content was positively correlated with electric conductivity, due to the dependable nature of electrical conductivity on honey moisture. The study area honey meets the basic honey quality standards both of the national and international honey quality specifications, except that the moisture content of croton honey which was some what out of the accepted range.

## Graphical abstract

The study was conducted to identify and characterize the monofloral honey types in Gesha‐Sayilem forest and the study area honey meets the basic honey quality standards both at the national and international honey quality specifications.

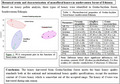

## 1 INTRODUCTION

Honeybees (*Apis mellifera* L.) obtain nectar and pollen from flowering plants (Bareke & Addi, 2019a). The availability of bee flora is the most important factor that can influence the activities of honeybees and their production (Keasar & Shmida, 2009). Knowledge of the availability of major honeybee forage species and their flowering calendar is very important to boost honey by predicting the frequency of honey harvest, honey flow period, and seasonal colony management (Bareke & Addi, 2018). A flowering calendar is a time table that indicates the approximate date and duration of the blossoming periods of the important nectar and pollen source plants (Bareke & Addi, 2018). Honey is defined as a sweet substance made by honeybees from the flowers of the plants or from parts of the plants or exudates of other insects which bees gather and transform by adding specific substances of their own, store by removing water, and leave in the honey comb to ripen (Codex Alimentarius Committee on Sugars, 2001; Ahmad and Shah, 2014). The variability of honey types produced in a particular area depends upon the diversity of nectar source plants (Addi & Bareke, 2019; Sibel & Mustafa, 2007). Honey types can be identified using the method of honey pollen analysis (melissopalynology). Melissopalynology is the study of the botanical and geographical origin of honey through microscopic analysis of honey sediments in honey samples (Louveaux et al., 1978). Each plant species has their own genetic code of inheritance and special structural patterns, which enable pollen grains of one species to be differentiated from another (Bareke & Addi, 2019b; Chauhan & Trivedi, 2011). Honey is produced either from multifloral or unifloral/monofloral. Harvesting of pure unifloral honey may not be very common. The term unifloral/monofloral honey is used to describe honey in which the major part of the nectar is derived from a single plant species (Legesse, 2013).

The physicochemical properties of honey are influenced by the nectar types that the honeybee used, climatic and soil type, and postharvest honey‐handling practices (Belay et al., 2013). There are variations in physicochemical properties among monofloral honey of different botanical origins (Legesse, 2013; Makhloufi et al., 2009; Ahmad and Nanda, 2015a). The physicochemical properties and pollen spectrum of honey have been used to authenticate unifloral honey (Anklam, 1998; Louveaux et al., 1978).

Multivariate analysis of honey using principal component analysis (PCA) has been widely used in classification of honey types based on honey pollen analysis (Ahmad et al., 2015b; Bareke & Addi, 2019b), which is part of honey quality parameters to characterize honey and to trace back botanical origin of honey. Characterizing honey by its dominant plant origin is very common and has been used to stimulate the type of honey for the market (Felsner et al., 2004).

Southwestern parts of the Ethiopia have an immense potential for honey production due to the availability of diversified natural vegetation and agroforestry (trees and shrubs). Because of the availability of natural plant species, large volume of honey is produced annually in the area. So far, there is limited information about the botanical origin and physicochemical analysis of honey produced in the study area. Beekeepers in Gesha‐Sayilem forest have a better understanding of the value of the forest for honey production. The honey production system is dominated by the traditional beekeeping system, and honey produced from the traditional hive is poor in quality.

Although honey is widely produced and consumed in this area, there is no information reported on the physicochemical properties of monofloral honey harvested from the Gesha‐Sayilem forest in Kaffa Zone, southwest Ethiopia. The quality of honey is mainly determined by its sensorial, chemical, physical, and microbiological characteristics (Gomes et al., 2010). Therefore, this study was conducted with the objectives of identifying and characterizing the monofloral honey types found in the Gesha‐Sayilem forest.

## 2 MATERIALS AND METHODS

### 2.1 Description of the study area

**FIGURE 1:**
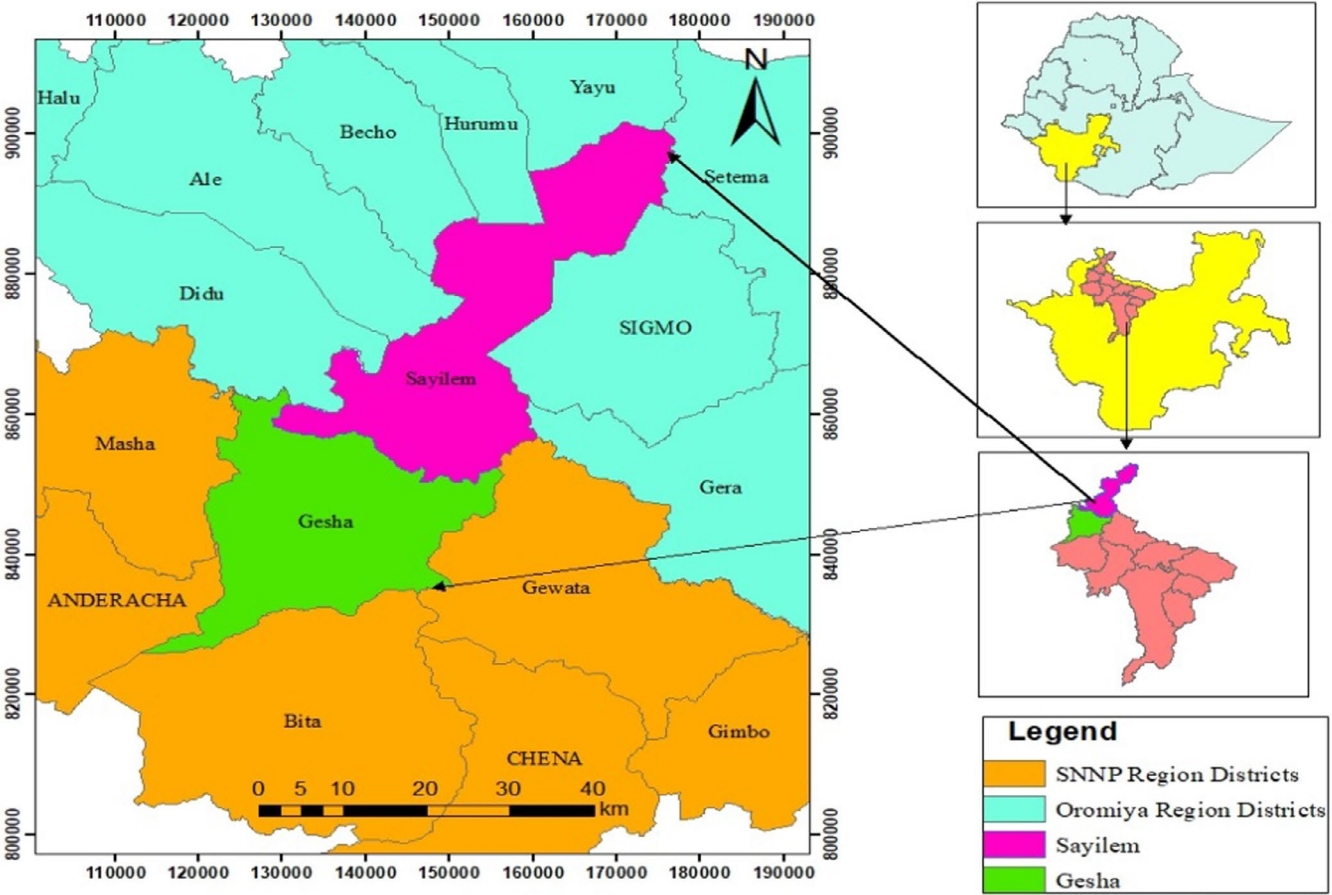
Location map of Gesha and Sayilem districts (Addi et al., 2020)

This study was conducted in the two districts of Gesha and Sayilem in Kaffa Zone of the Southern Nations Nationalities Peoples Regional State (Figure 1). Gesha district is geographically located between 7^0^ 35.36 N latitude and 35°, 4,527 E longitudes, and Sayilem is located between 7^0^ 49 57 N latitudes and 35^0^49.32 E longitudes. The southern part of Gesha district is bordered by Bita district in the west by the Sheka Zone, in the north by Illubabor Zone of Oromia region, and in the east by Gewata district. The topography of the landscape is undulating, with valleys and rolling plateau, and some with flat plains.

### 2.2 Determination of the botanical origin of honey

A total of 15 crude honey samples were collected during honey flow seasons from February to March for *Vernonia* honey, April to May for *Schefflera* honey, and May to June for *Croton* honey. For each honey type, five samples weighing 500 g were purchased. The honey samples were strained using double sieves and cheese cloth at the Holeta Bee Research Center (HBRC) laboratory to get pure honey from crude honey. The honey samples were then stored at 4℃ for further analysis (Bareke & Addi, 2019b). For honey pollen analysis, the method recommended by the International Commission for Bee Botany (Louveaux et al., 1978) was adopted. Pollen types were identified by comparing with reference slides of pollen collected directly from the plants in the study area.

For quantification of the pollen types, at least 500 pollen grains were counted from each sample (Oliveira et al., 2010). The percentage frequency of the pollen taxa in all the samples was calculated excluding polleniferous plant species which were observed during honey pollen analysis (Bareke & Addi, 2019b). Predominant pollen types (>45%) of the total pollen grains were counted; secondary pollen types (16%‐45%), important minor pollen types (3%‐15%), and minor pollen types (3%) were types of pollen allocated to one of the frequency classes for nectar source plants (Louveaux et al., 1978). Honey with predominant pollen types was considered monofloral.

### 2.3 Analysis of physicochemical properties of forest honey

The honey quality was determined based on the harmonized methods of the International Honey Commission (Bogdanov, 2009). The major parameters that were considered in the analysis were moisture content, electrical conductivity, hydroxymethylfurfural (HMF), PH, free acidity, enzymes (invertase and proline), and sugars (sucrose, fructose, and glucose). The moisture content of honey samples was determined using an Abbé refractometer that can be adjusted at 20℃ and regularly calibrated with distilled water. The honey samples were homogenized and placed in a water bath until all the sugar crystals were dissolved. The electrical conductivity of honey was determined following the procedure of Codex Alimentarius Commission Standards (Codex Alimentarius Committee on Sugars, 2001). The electrical conductivity of the honey was measured based on the electrical conductance of the sample using a conductivity meter. pH of the honey was determined using the methods of Chatway (1978). For the determination of pH of 10 gm of honey was mixed with 75 ml of distilled water in a 250‐ml beaker. The solution was stirred using a magnetic stirrer, and the pH electrodes were immersed in the solution, and the pH was recorded. For the determination of the hydroxymethylfurfural, five grams (5 g) of honey was weighed and mixed with 25 ml of distilled water. The solution was filtered using a Whatman paper, and the reading was made using HPLC equipped with UV detection.

### 2.4 Determination of sugar

The standard of fructose, glucose, and sucrose was purchased for determination of sugar in honey. About 5 g of honey was weighed into the beaker and dissolved in 40 ml water. 25 ml of methanol was pipetted into a 100‐ml of volumetric flask, and the honey solution was transferred to the flask. The methanol mixture of the honey solution was filtered using filter paper, and the solution is poured in vials and stored as the standard solution for the determination of sugar using high‐performance liquid chromatography (HPLC). The invertase and proline content of honey types were determined following the method recommended by the International Honey Commission (Bogdanov, 2009).

### 2.5 Data analysis

Data were analyzed using one‐way ANOVA. Turkey's multiple comparison test was applied at the significance level of 0.05, the PCA component plot was constructed using R software.

## 3 RESULTS AND DISCUSSIONS

### 3.1 Botanical origin forest honey

**FIGURE 2:**
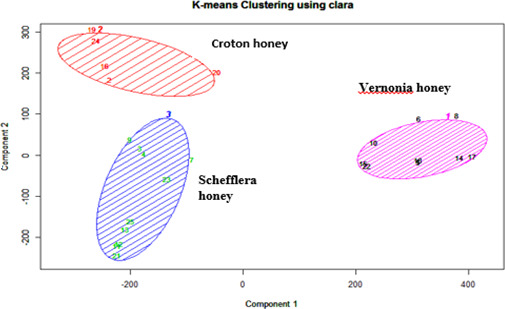
PCA component plot in the function of floral origin of honey

Based on honey pollen analysis, honey samples were classified botanically into two categories: forest plants and nonforest plants. The majority (86.7%) of honey samples were obtained from forest trees, while only 13.3% were from weeds and crops, and this is because a major portion of the area was covered by natural forest trees and shrubs. The classification of pollen percentage count using silhouette and PCA clustering indicated that three types of honey were identified, such as *Schefflera abyssinica* honey type, *Croton macrostachyus* honey type, and *Vernonia amygdalina* honey type. Honey from *Schefflera abyssinica* and *Croton macrostachyus* were found at the right side of PCA plots, and *Vernonia amygdalina* was found on the left side of the PCA Figure 2.

*Schefflera abyssinica* contributed more than 60% of the pollen count. The dominance of pollen from *Schefflera abyssinica* was attributed to widespread distribution in moist highlands of southwestern and southeastern parts of Ethiopia. *Schefflera abyssinica* honey is considered to be monofloral honey, and it is extra white with a characteristic aroma and very pleasant taste (Belay et al., 2013). The honey contains very little amount of pollen and is easy to strain; thus, when it is in the liquid state, it is clear and transparent. The honey is commonly harvested after the flowering period of the plant, which takes place between April and May. *Croton macrostachyus* honey was the second dominant honey in the mid‐altitude areas of the forests. The crystallization of the honey is very coarse with a rough and sandy texture. Because this honey is harvested during rainy season, the moisture content is also relatively high. The main harvesting time for croton honey is between mid‐May and June. *Vernonia* honey is the third dominant honey obtained mainly from *Vernonia amygdalina*. However, different species of *Vernonia* spp including *Vernonia auriculifera*, *Vernonia thomsoniana*, *Vernonia schimperi*, *Vernonia rueppellii*, and others are contributing to the production of *Vernonia* honey. *Vernonia* honey is mostly harvested at mid‐altitude and grown as secondary vegetation, where there are high disturbances at the edge of natural forest. In some localities, there is an overlapping of the flowering period with *Coffea arabica*. As a result, there is some degree of mixing of *Vernonia* honey with Coffee honey. *Vernonia* honey is very dark in color even after crystallization, and it tends to granulate uniformly. The honey has a very strong flavor and bitter taste, and traditionally, *Vernonia* honey is well‐known for its medicinal property. Vernonia honey is commonly harvested between the months of February and March.

### 3.2 Physicochemical properties of Gesha‐Sayilem forest honey

The results of the physicochemical analysis of the monofloral honeys for *Schefflera abyssinica*, *Croton macrostachyus*, and *Vernonia amygdalina* are presented in Table 1.

**TABLE 1:** Physiochemical properties of forest honey of Gesha‐Sayilem forest

Parameter	*Vernonia amygdalina*	*Schefflera abyssinica*	*Croton macrostachyus*
Moisture content (%)	18.3 ± 1.10a	18.10 ± 1a	21.30 ± 1.05a
HMF	1.2 ± 0.10b	2.36 ± 0.15a	2.56 ± 0.15a
Invertase	113.6 ± 1.65c	181.90 ± 1.48a	126.60 ± 0.90b
Proline	210.6 ± 1.67b	197.60 ± 1.25b	827.30 ± 1.2a
pH	4.05 ± 0.04a	3.97 ± 0.015a	4.00 ± 0.50a
Free acidity	7.00 ± 1.00b	4.00 ± 1.0c	10.00 ± 1a
EC	0.20 + 0.16b	0.087 + 0.03c	0.24 ± 0.02a
Fructose	38.63 ± 0.96a	39.86 ± 0.92a	37.1 ± 1.05b
Glucose	35.66 ± 2.2a	35.74 ± 0.47a	36.4 ± 0.85a
Sucrose	3.16 ± 0.06b	4.8 ± 0.1a	3.2 ± 0.06b
F + G	74.26 ± 0.95a	73.86 ± 0.81a	73.71 ± 0.85a

Note

Different letters show significant differences.

a, b denotes two papers published in the same year at different time

Abbreviations: EC, electric conductivity; F, fructose; G, glucose; HMF, hydroxymethylfurfural.

### 3.3 Moisture content

The mean moisture content of the honey samples of *Vernonia amygdalina* honey was 18.3 ± 1.02 g/100 g (17.1–19.1 g/100 g), that of *Schefflera abyssinica* honey was 18.1 ± 1 (17.1–19.1 g/100 g), and that of *Croton macrostachyus* honey ranged from 19.2 to 23.3 g/100 g with the mean value of 21.2 ± 1.05 g/100 g (Table 1). The moisture content of *Vernonia amygdalina* and *Schefflera abyssinica* honey types was found to be within an accepted range of Ethiopian honey quality standards (Adgaba, 1999). However, the moisture content of *Croton* honey was relatively higher than the accepted range. This is because the honey‐harvesting time of this species coincides with the short rainy season and hygroscopic properties of honey. The variation of the moisture content of honey among different honey source plants is due to harvesting of unripe honey, unsuitable honey storage containers and storage places, and humidity of the air surrounding the beehives (Ewa et al., 2019; Fechner et al., 2016). The mean moisture content of honey was also higher than that of the country's average 20.6 g/100 g (Adgaba, 1999). The maximum limit for moisture content set by the International Honey Commission is 20 g/100 g (IHC, (2002)). In Europe, in many national beekeeping organizations such as Germany, Belgium, Austria, Italy, and Spain set out the moisture content of maximum values of 17.50–18.50 g/100 g for special classes of quality honey, whereas for Switzerland, it was less than 20 g/100 g is acceptable (Bogdanov et al., 1999). According to the Ethiopian standard, the moisture content of the honey grouped into “Grade A,” 17.50–19.00 g/100 g; “Grade B,” 19.10–20.00 g/100 g; and “Grade C,” 20.10–21.00 g/100 g. *Vernonia amygdalina* and *Schefflera abyssinica* honeys can be grouped as “Grade A” honey based on the Ethiopian standard (2005), whereas *Croton* honey can be grouped as “Grade C.”

### 3.4 Hydroxymethylfurfural

The HMF value of the *Vernonia* honey ranged from 1.1 to 1.3 mg/kg with a mean value of 1.2 ± 0.1 mg/kg, that of *Schefflera abyssinica* honey ranged from 2.2 to 2.5 mg/kg with the mean value of HMF 2.3 ± 0.15 mg/kg, and that of *Croton* honey from 2.4 to 2.6 mg/kg and mean value of 2.56 ± 0.15 mg/kg (Table 1). The HMF of *Vernonia* honey was significantly different from that of *Schefflera* honey and *Croton* honey. The value of HMF in the above honey types was found within an acceptable range of Ethiopian honey qualities set by Ethiopian quality control agencies and International honey quality standards of EU and Codex Alimentarius (CA). According to the HMF content criteria, the shelf‐life of honey is set by national regulations worldwide. Based on this criterion, the packaging plants are obliged to comply with the requirement when establishing the use by date to be printed on their labels (Belay et al., 2013). Fresh honey does not contain HMF (Bogdanov et al., 2004) or contain only traces of HMF, which is an important criterion for the evaluation of storage time and heat damage (Ruoff & Bogdanov, 2004 and Ahmad and Nanda, 2016).

### 3.5 pH

The pH of the *Vernonia* honey ranged from 4.01 to 4.1, with a mean value of 4.05 ± 0.04; that of *Schefflera* honey ranged from 3.95 to 3.98, with a mean value of 3.97 ± 0.15; and that of C. *macrostachyus* honey ranged from 3.5 to 4.5, with a mean value of 4 ± 0.5 (Table 1). There was no significant variation in pH between the three types of honey, and the honey types were found to be acidic. All the three types of honey (*Vernonia*, *Schefflera*, and *Croton*) are found to be acidic, with a pH value ranging between 3.9 and 4.5. This is due to the presence of organic acids that contribute to honey aroma and stability against fermentation by microorganisms (Belay et al., 2013). The mean pH value of the honey from the study area is in line with the world standard, between 3.2 and 4.5 (Bogdanov et al., 1999).

### 3.6 Free acid

The mean free acidity content of the *Vernonia amygdalina* honey samples was 7 ± 1 meq/kg (6–8 meq/kg) and that of *S*. *abyssinica* honey was 4.1 ± 1, with the range of 3–5 meq/kg. For *C*. *macrostachyus*, the mean free acid content was 10 ± 1 (9–11 meq/kg) (Table 1). There was a significant difference in the free acid content of honey samples due to the botanical origin of honey and sampling locations (*p* < .05). The mean free acid content of the Harenna forest honey samples was 34.57 ± 4.80 meq/kg (25.49–48.81 meq/kg) (Belay et al., 2013). The mean free acid value of the current study was below the national average, 39.9 meq/kg (Adgaba, 1999), and satisfied the CA, EU, and Ethiopian standards. According to Küçüka et al., (2007), a lower value of acid indicates the absence of undesirable fermentation. The maximum limit for free acid set by the CA is 50 meq/kg of honey and that by the EU and Ethiopian standard is 40 meq/kg of honey. Free acidity of honey is due to the presence of organic acids and inorganic ions such as the gluconic acid with their lactones or esters, phosphate, and chloride (Belay et al., 2013). In addition to this, due to floral origin or variation in harvesting season, there is variation in free acidity of honey (Perez‐Arquillué et al., 1994). Free acid measurement is useful for evaluation of honey fermentation, authentication of unifloral honeys, and differentiating nectar of flora from honeydew honeys (Nandaa et al., 2003).

### 3.7 Electrical conductivity

The electrical conductivity of *Vernonia* honey was varied from 0.13 to 0.2 mS/cm, with a mean of value of 0.20 ± 0.16. For *Schefflera abyssinica*, the mean EC was 0.087 ± 0.03 mS/cm (0.07–0.09 mS/cm), and for croton honey was 0.24 ± 0.02 mS/cm, with the range of 0.23–0.26 mS/cm. Electrical conductivity depends on ash, organic acids, proteins, some complex sugars, and polyphenols contents and varies with botanical origin. The electrical conductivity of the honey analyzed for this study was found to be less than 0.8 mS/cm^−1^, which is an indication of nectar honey. Thus, electrical conductivity of honey samples in the Gesha‐Sayilem forest was found to be within an international range, with the maximum limit of 0.8 mS/cm for most nectar honey. Thus, the honey of the study forest satisfied the Codex Alimentarius and EU standards.

### 3.8 Invertase and proline

The mean value of the invertase content of *Vernonia* honey was 113.6 ± 1.6 (112.3–115.5), that of *Schefflera* honey was 181.9 ± 1.48 (180.3–183.2), and that of Croton honey was 126.6 ± 0.90 (125.7–127.5). There was significant difference (*p* < .05) between the three honey types or invertase contents.

The mean value of the proline content of *Vernonia* honey was 210.6 ± 1.67 (209.2–212.4), and the average proline content of *Schefflera* honey was 197.6 ± 1.25 (196.5–198.8). The mean value of proline content of *Croton* honey was 827.3 ± 1.2 (826.2–828.5). There was a significant difference (*p* < .05) between honey types in proline content. The enzyme level of the honey is an important parameter affecting the physicochemical properties of honey. There was significant difference (*p* < .05) between the honey types in invertase and proline contents. This could be due to the differences in botanical and geographical origin of honey.

### 3.9 Sucrose

Analysis of sucrose content is used to detect the adulteration of honey with table sugar or to determine the amount of sucrose found naturally in a given honey sample. The mean sucrose content of *Vernonia* was 3.16 ± 0.06 g/100 g, that of *Schefflera abyssinica* was 4.8 ± 0.1 g/100 g (4.7–4.9 g/100 g), and that of croton honey was 3.2 ± 0.06 (3.22–3.3392 g/100 g). The level of sugar content in honey varies according to the origin of the nectar source plants, and it is used to identify adulteration in honey by adding sugarcane or other sugars. The mean sucrose content of analyzed honey samples of *Vernonia*, *Schefflera*, and *Croton* honeys ranged between 3.16 and 4 g/100 g. The mean apparent sucrose content of the honey was lower than the national average of 3.6% (Adgaba, 1999). The result revealed that the honey produced from Gesha‐Sayilem forest was natural and not adulterated and satisfied the Codex, EU, and the Ethiopian standards. The CA, EU, and Ethiopian standards have a maximum limit of 5 g/100 g of honey (Bogdanov, 2001).

### 3.10 Fructose and glucose

Analysis of the fructose content of honey for the three types of honey showed that croton honey was significantly different (*p* < .05) from *Schefflera* and *Vernonia* honey. The mean value of the fructose content of *Vernonia* honey was 38.63 ± 0.96 and that of *Schefflera* and *Croton* honeys was 39.86 ± 0.92 and 37.1 ± 1.05, respectively. Similarly, the glucose content in *Vernonia amygdalina* and *Schefflera abyssinica* honey was 35.66 ± 2.2 and 3,574 ± 0.47, respectively, and the mean glucose content of *Croton* was 36.4 ± 0.85. The sum of fructose and glucose of the three honey types was above 73 (Table 1). According to the Codex standard for honey (2001), the fructose and glucose content of honey is >60 g/100 g. Thus, this result is found to be in acceptable range.

### 3.11 Correlation between the physicochemical properties of honey

Pearson's correlation is used to show if there is a linear relationship between two sets of parameters. Accordingly, the Pearson correlation coefficients between all parameters used for physiochemical properties of honey were presented in Table 2. There was a significant strong correlation between proline and free acid (*p* < .01), and invertase and sucrose (*p* < .01). Moisture content was positively correlated with Electric conductivity (*p* < .05). This could be due to the dependable nature of electrical conductivity on honey moisture. This was supported by Bogdanov, (2001) and Belay et al., (2013) by adding water to honey, all of its physical properties showed remarkable changes, with major changes occurring in conductance. Pure honey was characterized by a low conductance value. If honey is adulterated with water or saturated sugar solutions, it will display greater conductance than pure honey. There was negative correlation between the moisture content of honey with sugars (sucrose and fructose). The higher moisture content of honey affects the level of sugars, leading to fermentation of honey.

**TABLE 2:** Pearson correlation coefficients among the analyzed parameters

Parameter	Moisture	HMF	Invertase	Proline	pH	Free acid	EC	Fructose	Glucose	Sucrose
Moisture	1	428	−0.323	0.737^b^	−0.23	0.673^b^	0.791^b^	−0.641	0.298	−0.424
HMF		1	0.525	0.588	−0.093	119	0.128	−0.152	0.256	0.426
Invertase	−0.323	0.525	1	−0.354	−0.111	−0.721^b^	−0.725^b^	0.589	−0.071	0.988^b^
Proline				1	−0.015	0.830^b^	0.797^b^	−0.727^b^	0.275	−0.469
pH					1	0.228	−0.006	−0.377	0.175	−0.084
Free acid						1	0.939^b^	−0.908^b^	0.113	−0.804^b^
EC							1	−0.848^b^	0.134	−0.799^b^
Fructose								1	−0.359	0.667^b^
Glucose									1	−0.069
Sucrose										1

Abbreviations: EC, electric conductivity; HMF, hydroxymethylfurfural.

Correlation is significant at the 0.05 level (2‐tailed).

^b^ Correlation is significant at the 0.01 level (2‐tailed).

## 4 CONCLUSION

The pollen analysis of honey indicated that *Schefflera abyssinica*, *Croton macrostachyus*, and *Vernonia amygdalina* were the major sources of monofloral honey in Gesha‐Sayilem forest due to their abundance and high nectar yielding potential of these species. The moisture content of *Vernonia amygdalina* and *Schefflera abyssinica* honey types was found to be within an accepted range of Ethiopian honey quality standards. However, the moisture content of *Croton* honey was relatively higher than the accepted range due to overlapping of the honey harvesting time of the species with short rainy season. All the three types of honey (*Vernonia*, *Schefflera*, and *Croton*) were found to be acidic, with a pH value ranging between 3.9 and 4.5, which is found within the acceptable range. The electrical conductivity of the three honey types in the Gesha‐Sayilem forest was found to be within an international range, with the maximum limit of 0.8 mS/cm for most nectar honey. There is significant strong correlation between proline and free acid. Moisture content is positively correlated with electric conductivity. This could be due to the dependable nature of electrical conductivity on honey moisture. The study area honey meets the basic honey quality standards both at the national and international honey quality specifications, except the moisture content of croton honey which is somewhat out of the accepted range.

## DATA AVAILABILITY STATEMENT

As the authors of this manuscript we agreed that Data will be available from the corresponding author upon reasonable request provided that the data are not publicly available due to privacy or ethical restrictions.
